# Approaches to support continued iCCM implementation during a flooding emergency in rural Bangladesh

**DOI:** 10.7189/jogh.09.021001

**Published:** 2019-12

**Authors:** Rashed Shah, Nathan P Miller, Golam Mothabbir

**Affiliations:** 1 Save the Children US, Washington, D.C., USA; 2 UNICEF, New York, New York, USA; 3 Save the Children International, Dhaka, Bangladesh

## Abstract

**Background:**

More evidence is needed on how integrated community case management (iCCM) service delivery is affected and on how to maintain service availability during crises. This study documented the implementation of iCCM through two cadres of community health workers (community health care providers [CHCPs] and village doctors [VDs]) in communities that were affected by a 2015 flooding emergency in Bangladesh.

**Methods:**

We conducted a retrospective case study to assess iCCM services provided by CHCPs and VDs during a flooding emergency that occurred from June to August 2015. We purposively selected nine unions within four sub-districts in Bhola District. In this mixed methods study, we analyzed trends in quantitative service delivery indicators over the time period from January 2015 to February 2016. Qualitative data were obtained through 28 in-depth interviews and 13 focus group discussions with policy makers, implementers, supervisors, CHCPs, VDs, community leaders, and caregivers of under-five children.

**Results:**

All stakeholders reported disruptions in iCCM service delivery and in access to CHCPs and VDs for community members. The quantitative data showed a 30% reduction in average number of children who received treatment from both CHCPs and VDs during flooding months compared to pre-flood months (from 2273/month to 1593/month). There was also an increase in the number of children referred by CHCPs and VDs, reduced supervision, and increased stock-outs of commodities during the flooding months. CHCPs and VDs, in collaboration with community members, came up with several locally adapted initiatives to maintain iCCM services, including changing clinic hours according to the tide, organizing temporary clinics at alternative sites that were located on higher ground, use of community boats to visit clients in their homes, and use of mobile phones for communication with supervisors and community members.

**Conclusion:**

Our study results demonstrate that iCCM services can continue during a natural disaster, albeit with significant disruptions. Ad hoc adaptations to services by local implementers and community members were key in maintaining availability of services during the emergency. In future emergencies, service delivery could be significantly strengthened by enacting key preparedness activities prior to a natural disaster such as severe flooding.

Pneumonia, diarrhea, and malaria are the leading infectious causes of death among children under five years of age [1]. Despite reductions in under-five mortality globally, 5.6 million children under five years of age died in 2016, from mostly preventable causes [2]. A key reason for continued high rates of mortality in many low-income countries is lack of access to primary care for much of the rural population [3,4]. Integrated community case management (iCCM) has been increasingly promoted as a strategy to increase access to life-saving therapies for the most common childhood illnesses (eg, childhood pneumonia, diarrhea, and malaria) [5].

With an increased burden of morbidity and mortality and decreased access to care in emergencies, there is an interest in implementing iCCM at a larger scale in countries affected by conflict or natural disaster [6]. There have been several experiences with implementation of iCCM in emergencies, both in conflict settings and natural disasters [7-11]. However, more evidence is still needed on how service delivery is affected by crises, how to maintain service availability during a crisis and on the impact of iCCM services during emergencies.

Due to its geographical location, Bhola District, in Barisal Division, Bangladesh is particularly exposed to heavy rainfall, cyclones, and floods on an annual basis. With an approximate population of 1.7 million and about 200 000 children under five years of age, it consists of seven sub-districts (locally called upazilas), which are further divided into 70 unions (the smallest administrative unit). June, July, and August are the peak monsoon months, characterized by torrential rain.

In May 2015, a nor’wester, along with hailstorm and lightning, caused heavy damage in Bhola and several other districts in Bangladesh. Local newspapers [12-14] and the Network of Information, Response and Preparedness Activities on Disaster (NIRAPAD, a local network of 22 Bangladeshi non-government organizations who work for reducing disaster risk in Bangladesh) published reports [15-18] on torrential rains, monsoon floods, riverbank erosion, and embankment collapse in Bhola District during June-August, 2015. Tropical cyclone Komen hit in July, 2015, and affected more than 1.5 million people, several thousand homes were flattened or flooded across the coastal areas of Bangladesh, including Bhola District [19].

From February 2012 to September 2014, Save the Children (SC) supported the Ministry of Health and Family Welfare (MOH&FW) to implement iCCM, focusing on treatment of childhood pneumonia (with amoxicillin) and diarrhea (with ORT and zinc) among under-5 children in rural communities of four districts (out of six districts) in Barisal Division (Barisal, Bhola, Patuakhali, and Borguna). SC provided technical assistance, training, and supervisory support for two community-level health cadres, community health care providers (CHCPs), and village doctors (VDs). The project also ensured supply of necessary commodities (ARI timer, thermometer, cotton, field registers, field bag, pen, amoxicillin syrup, ORS, paracetamol tablet and syrup, gentian violet), counseling caregivers, supervision and monitoring of field activities (jointly with the MOH&FW), and reporting field service statistics using the district health information system (DHIS).

CHCPs are MOH&FW employees and provide services in community clinics (CCs), which are static rural health facilities that cover a catchment population of 6000 people. These CCs remain open six days a week from 9 am to 3 pm and provide thirty-one essential medicines. The CHCPs have at least a 12^th^ grade education and receive three months pre-service training focused on preventive and curative health services. CHCPs assess and treat children in the CC, provide childhood immunization, offer health counseling to caregivers and refer children with serious illnesses to union or sub-district health centers. CHCPs also conduct community mobilization and dissemination of health messages. Medical officers from the nearest union or sub-district health center supervise CHCPs.

CHCPs receive commodities through the government supply chain. Medicines and supplies are distributed from the national level to the sub-districts, with pre-determined allotments for each CC, and then are distributed from sub-districts to the CCs. Since the estimation of required medicine is done at the national level and does not take into account the varying populations served by different CCs, those that serve larger populations experience frequent drug stock-outs. As mentioned above, SC coordinated with MOH to ensure a consistent supply of necessary commodities to prevent drug stock-outs within the project area during non-flooding months.

VDs are private health care providers working independently. VDs mostly receive short training (from a few weeks to a few months) on common illnesses/conditions from semi-formal private institutions that are unregistered and unregulated and do not follow a standard curriculum. They have been providing health services, including iCCM services, either from drug shops/dispensaries or from their homes, for decades. They also make house calls and provide domiciliary services. Most of the villagers depend on the VDs for emergency health care needs when the CC is closed. Like CHCPs, VDs refer complicated patients to union or sub-district health facilities. The VDs do not receive any medicine or supply from the MOH&FW. Instead, they buy and maintain their own medicine stocks. Our study aimed to understand the experiences of implementing iCCM in communities that were affected by the 2015 flooding emergency in Barisal, and to document actions taken to adapt iCCM service delivery in the emergency context and to identify promising approaches for addressing bottlenecks in iCCM programs during future emergencies.

## METHODOLOGY

We conducted a retrospective case study in early 2016 to assess iCCM services provided by CHCPs and VDs during a flooding emergency that occurred from June to August 2015. The study locations included nine unions from four sub-districts (Sadar, Burhanuddin, Daulatkhan, and Tazumuddin) in Bhola District. These sub-districts and unions were selected purposively based on a range of factors including areas that were accessible to the study team, areas that were not heavily affected by seasonal economic migration and consideration of budget and time constraints. The names of selected sub-districts and unions and the number of affected CHCPs and VDs are enumerated in **Table 1**.

**Table 1:** Selected sub-districts and unions with number of affected CHCPs and VDs

Name of sub-district	Name of union	Total number of CHCPs	Number of CHCPs affected	Total number of VDs	Number of VDs affected
Bhola Sadar	Purbo Elishia	8	1		
	Razapur	4	1		
	Vaduria	4	1		
	Kachia	3	1	1	1
Burhanuddin	Bromanika	5	4	6	4
	Kutuba	4	1		
	Pokhia	2	1		
Doulatkhan	Sayadpur	7	2		
Tazumuddin	Chandpur	7	1		
**Total**		44	13	7	5

CHCP – community health care providers, VD – village doctors

### Quantitative methods

We retrieved available quantitative data from DHIS and field monitoring reports on the number of under-five children who were treated and referred by CHCPs and VDs, the proportion of CHCPs and VDs who had essential commodities and who received supportive supervision before, during, and after the flooding months. We analyzed these data to assess trends in key indicators over the time period from January 2015 to February 2016.

### Qualitative methods

#### Study tool development

UNICEF and SC, jointly in consultation with MOH&FW, developed the qualitative study tools, with separate tools for each category of respondent and data collection method. We conducted an initial pre-test prior to finalizing the data collection tools.

#### Data collection

Qualitative data collection was conducted during January – February 2016 by locally recruited university graduates. A total of six data collectors were split into three teams, with two in each team. Each team was supervised by one research coordinator. Data collectors received three days’ training on qualitative research methodologies and facilitation skills, using a mix of lectures and role-play sessions. Qualitative data were collected through semi-structured in-depth interviews (IDIs) and focus group discussions (FGDs), with each session conducted in the local language (Bangla). The types and numbers of participants for IDIs and FGDs are presented in **Table 2**.

**Table 2:** Distribution of respondents for IDIs and FGDs

Level of respondents	Respondents	IDI (#)	FGD (#)	Participants (#) in FGD
National	Policy makers and implementers (MOH&FW, SC, and UNICEF)	3		
District	Civil surgeon	1		
Sub-district	Supervisors	4		
Sub-district	Upazila Health and Family Planning Officers	3		
Union	Supervisors		2	8
Community	Caregivers of under-5 children		6	23
	Community leaders		5	18
Community	Community health care providers	12		
	Village Doctors	5		
	**Total**	**28**	**13**	**49**

FGD – focus group discussion, IDI – in-depth interview, MOH&FW – Ministry of Health and Family Welfare, SC – Save the Children

#### In-depth interviews

Semi-structured 60-minute IDIs were conducted with four categories of informants: 1) national level policy makers and implementers (MOH&FW, UNICEF, and SC), 2) program managers at the district and sub-district levels (eg, civil surgeon at the district and upazila health and family planning officers [UHFPO] in sub-districts), 3) supervisors in sub-districts (eg, health inspectors and project officers), and 4) iCCM service providers (eg, CHCPs and VDs) at the community level. We conducted 28 IDIs (12 with CHCPs, 5 with VDs, 8 with district and sub-district level managers and supervisors, and 3 with national-level policy makers). Interviews with policy makers focused on program successes, issues, and challenges in terms of policy, advocacy, and strategies at the national level to help the iCCM programs be more effective during crises. The IDIs with district and sub-district level program managers and supervisors focused on key technical areas of the program, while the interviews with CHCPs and VDs covered the major programmatic themes, including training and human resource management, supervision and service delivery, referral system, supply chain, communication and social mobilization, monitoring and evaluation, and health information systems, and how these functioned during the flooding emergency.

#### Focus group discussions

Separate 90-minute FGDs were conducted with union-level supervisors, community leaders, and caregivers of under-five children. A total of 13 FGDs were conducted and each FGD had four to eight participants. Discussions with union-level supervisors focused on the past and current situation of the iCCM program, opinions on their role in supporting the iCCM program, factors contributing to or limiting success of the program and suggestions for the future, and their experience during the floods. The FGDs with caregivers and community leaders explored the decision-making process for health care seeking, community participation in the iCCM program, and the community perceptions regarding the iCCM program. Finally, during these FGDs respondents described their experience with iCCM services during the flooding emergency and provided recommendations for improving the program during future flooding emergencies.

#### Data management and analysis

All IDIs and FGDs were audio recorded and transcribed verbatim with the help of expanded field notes. We used a combination of deductive and inductive approaches to analyze the data. First, we used the iCCM benchmarks [20] as an overall framework. Therefore, the highest level grouping of data was by the eight components of the iCCM benchmark framework: 1) coordination and policy setting, 2) costing and financing, 3) human resources, 4) supply chain management, 5) service delivery and referral, 6) communication and social mobilization, 7) supervision and performance quality assurance, and 8) monitoring and evaluation and health information systems. We then used inductive thematic analyses of data to identify sub-themes. Data were then re-organized according to the pre-existing and identified themes and sub-themes for final analysis, using Atlas.ti software (Scientific Software Development, Berlin, Germany) [21].

### Ethical approval

Informed consent was obtained from all individual study participants. This study received ethical approval from Bangladesh Medical Research Council. The SC ethical review committee also approved the study protocol.

## RESULTS

All study respondents described the devastating impact of flooding on the living conditions of the community, including food insecurity, and damage to roads and other infrastructure. Community members (caregivers and community leaders) were not warned about the flood nor were they prepared for this disaster. CHCPs asserted that children were the most affected victims of the floods. Respondents identified water-borne diseases (eg, diarrhea, dysentery, typhoid), pneumonia, skin disease, and malnutrition as important causes of morbidity following flooding. VDs also highlighted the adverse health effects attributable to lack of safe water, sanitation and hygiene, and lack of appropriate shelter during and following the floods. One VD narrated as below:

“At that time, many of the children suffered from ailments like fever, cold and cough, dysentery, headache, pneumonia, and diarrhea. In addition, there were also children suffering from malnutrition.”

### Effect of flooding on iCCM services

#### Access, treatment, and referral

All CHCPs mentioned the adverse effects of flooding on their service delivery. Likewise, several VDs reported that they had to close down their dispensary/workplace during flooding. One CHCP described the challenges to continue services at her CC:

“The main problem was running the clinic on schedule. Due to flood and tide water coming and submerging the CC in the morning hours, our tube-well went under water, and we had to open the CC at about 10 a.m. For the second tide water at around 1 p.m., we had to close the CC by that time. We had a small amount of time to work. Besides, due to high tide water, we had to pack and unpack everything every day. We were forced to put one table upon another and then a chair upon the table to keep important medicines and record books out of the reach of tide water every day and arrange everything for work once again the next morning.”

**Figure 1:**
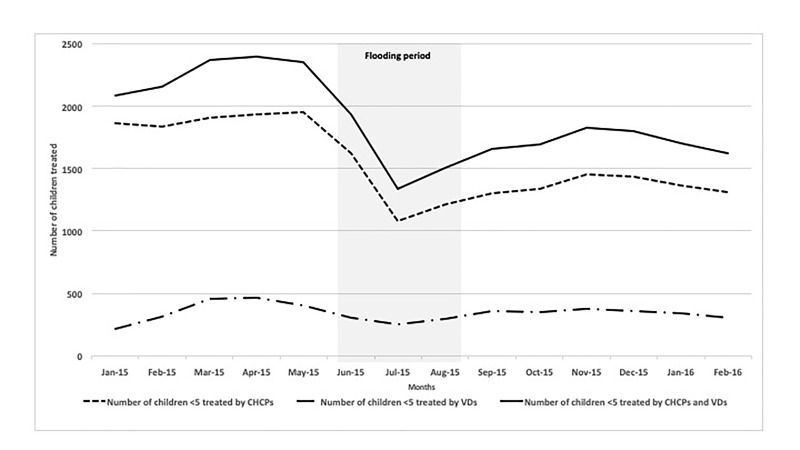
Number of children under 5 years treated by CHCPs and VDs, by month, in the flood-affected areas from January 2015 to February 2016.

Consistent with this qualitative finding, quantitative results also revealed a reduction in the number of children treated by CHCPs and VDs during flooding months. The total number of under-five children treated by both CHCPs and VDs declined from an average of 2273 per month during the five months prior to the flooding to as low as 1593 per month between June and August, the flooding months (a 30% reduction). The number of children receiving treatment by both CHCPs and VDs increased by 8% during the 6 months after the floods, with an average of 1716 children treated per month (**Table 3** and **Figure 1**).

**Table 3:** iCCM service indicators in flood-affected areas during the months before, during and after flooding

Indicator	Pre-flood period					Flooding period			Post-flood period					
	Jan-15	Feb-15	Mar-15	Apr-15	May-15	June-15	July-15	Aug-15	Sep-15	Oct-15	Nov-15	Dec-15	Jan-16	Feb-16
Number of children <5 treated by CHCPs	1866	1838	1911	1932	1948	1624	1081	1215	1304	1340	1452	1438	1364	1310
Number of children <5 treated by VDs	221	317	461	467	403	310	254	295	355	350	379	358	338	310
Number of children <5 referred by CHCPs	23	21	29	26	24	26	30	41	20	18	10	22	24	21
Number of children <5 referred by VDs	0	0	14	0	0	7	7	8	5	0	2	6	4	2
% of CHCPs who had essential commodities*	100	100	100	100	100	100	72.7	72.7	81.8	81.8	86.4	97.7	97.7	97.7
% of VDs who had essential commodities*	100	100	100	100	100	100	71.4	71.4	71.4	85.7	100	100	100	100
% of CHCPs received supportive supervision†	97.7	97.7	97.7	97.7	97.7	97.7	72.7	72.7	81.8	81.8	86.4	86.4	86.4	86.4
% of VDs received supportive supervision†	100	100	100	100	100	100	28.6	28.6	57.1	57.1	71.4	85.7	85.7	85.7

CHCP – community health care providers, VD – village doctors

*Essential commodities include ARI timer, thermometer and essential medicines (amoxicillin syrup, ORS, paracetamol tablets and syrup).

†At least one supervisory visit per month.

**Figure 2:**
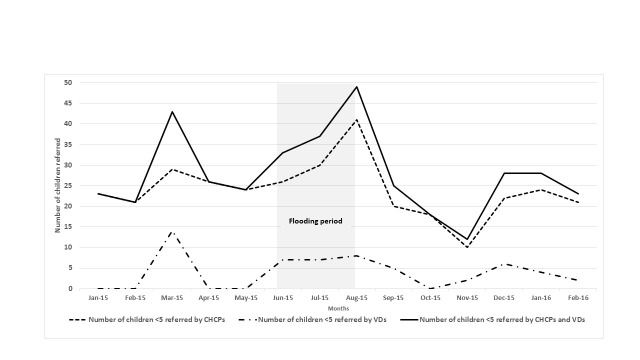
Number of children under 5 years referred by CHCPs and VDs, by month, in the flood-affected areas from January 2015 to February 2016.

The quantitative results reveal an increased number of children referred by CHCPs and VDs during flooding months. The average number of under-five children referred by both CHCPs and VDs increased 48%, from 27 per month during pre-flood months to 40 per month during flooding months (**Table 3** and **Figure 2**). However, despite the increased number of referrals, both CHCPs and community members highlighted challenges in completing referral during the floods. Several CHCPs described the difficulties for caregivers to travel to a sub-district hospital (which is often the referral site for CHCPs) because of flooded, destroyed, and damaged roads, as well as the financial burden associated with travel. One CHCP explained:

“Those whom I referred didn’t want to go to the referral hospital. They had problems with transport since the roads were all under water.”

Community members also talked about challenges to access the hospital due to submerged roads and lack of transportation.

“During the course of the flood, we had challenges with referral since the family sometimes had no option to travel to the referred hospital because all the roads were under water and the available options to move to the hospital were highly expensive.”

#### Supply of commodities

Transporting commodities from the sub-district to CCs was a major challenge during the flood. Due to the transportation challenge, coupled with a higher number of children with adverse health issues, several CHCPs reported stock-outs. Some CHCPs explicitly mentioned that transportation difficulties and high demand caused medicine shortages.

“Our problem was in carrying medicine from the sub-district hospital to our CC. As the roads were underwater, we could not use vehicles for transporting them. We had to carry medicine from the sub-district up to the Parishad building and then we had to bring the medicine from Parishad to the CC every day and take it back. This was very troublesome.”

“Demand was high due to an increased number of patients during that period. It would better if we could receive an increased amount of medicine supply…I needed more supply of paracetamol syrup, amoxicillin syrup, and metronidazole to combat the higher number of children with pneumonia, cold and cough, fever, dysentery, and so on.”

**Figure 3:**
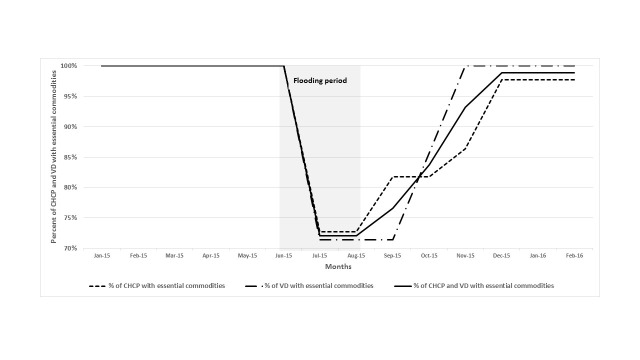
Proportion of CHCPs and VDs who had essential commodities*, by month, in the flood-affected areas from January 2015 to February 2016. *Essential commodities include ARI timer, thermometer, and essential medicines (amoxicillin syrup, ORS, paracetamol tablet and syrup).

The quantitative data show a reduction in the proportion of CHCPs and VDs who had essential commodities (ARI timer, thermometer, amoxicillin syrup, ORS, paracetamol tablet and syrup). About 82% of CHCPs and 81% of VDs had all essential commodities during the flooding months (June – August), while 100% of them had those commodities in possession without stockout in the months before the flood (January – May) and on average 91% of CHCPs and 93% of VDs had the commodities during the post-flood months (September 2015 – February 2016, **Table 3** and **Figure 3**).

#### Supervision

**Figure 4:**
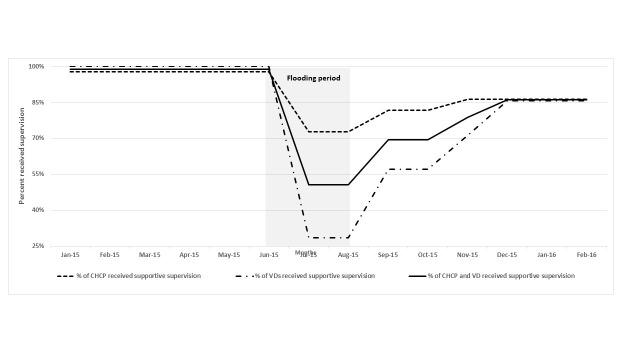
Proportion of CHCPs and VDs who received supportive supervision*, by month, in the flood-affected areas from January 2015 to February 2016. *At least one supervisory visit per month.

The difficult transportation during the flood period resulted in reduced field supervision. Program managers and supervisors at sub-district and union levels reported the difficulties they suffered due to the non-functioning transportation system during the flood. The quantitative data also show a reduction in the proportion of CHCPs and VDs who received supportive supervision during the flood. During pre-flood months, on average, 98% of CHCPs and 100% of VDs received supportive supervision at least once per month from their respective supervisors. The proportion of CHCPs and VDs supervised fell to 81% and 52% respectively during the flooding months. During the post-flood months, supervision for CHCPs and VDs increased to 85% of CHCPs and 74% of VDs (**Table 3** and **Figure 4**).

### Locally adapted action to continue delivering services

The CHCPs and VDs, in collaboration with community members, came up with several locally adapted initiatives to maintain iCCM services. Their initiatives included temporary clinics organized by CHCPs at alternative sites, such as at schools or local residents’ houses that were located in areas higher than the flood water level. They communicated the new clinic locations to community members in person or using loudspeakers at mosques or other central locations.

“I took the permission to sit in a specific house [to provide services] and informed the community through the speaker system. I also told them that if anyone sees any of their neighbors who are sick, they must tell their neighbor about this arrangement.”

In some instances, the community and the CHCP worked together to make treatment and referral possible. CHCPs obtained boats donated by community members for their movement from one location to another and to reach community members.

“The community arranged a small boat for me so that I could move or go to the place where there was an immediate need.”

The CHCPs also coordinated with the community for raising funds through donations from well-off community members and for arranging transportation for referred children. One CHCP describes the combined effort of the CHCP and the community for the referral service.

“We raised an amount of money from the donations of the community members and set up a welfare fund and a bank account. Those who are influential and well-off in the village helped us in whatever way possible.”

CHCPs also changed clinic service hours according to the tides by providing services during the low tide in the late morning and closing by the mid-afternoon, when the water would rise again. Additionally, they stored all items in a safe place at the end of the day to avoid water damage.

VDs covered the service gaps as they offered services from their dispensaries/workplace during the evening hours when the CC was already closed. In some instances they relocated their service delivery location, and sometimes they used makeshift rafts made of banana trees to reach their clients.

While constrained by damaged and submerged roads and lack of transportation during the flood, CHCPs tried to continue their efforts to convey health education messages to their respective communities and made changes to the content of health education messages. During the flood, most of the CHCPs emphasized health messages regarding water safety, sanitation, hygiene, diarrhea, and snakebites.

### Recommendations from respondents

Policy makers recommended ensuring integrated activities with multi-sectoral stakeholders (eg, health, education, local government, public health engineering) under the aegis of the disaster management and preparedness program during emergencies, as evidenced from one national level policy maker’s quote:

“Planning to continue iCCM services during flooding months in affected districts should be part of the emergency preparedness and planning. We [the health directorate] must establish effective collaboration and partnership approach with the district emergency preparedness cell and we also should focus on necessary planning in advance to ensure continued supply of commodities to service providers and facilities in the areas those have potential risk of supply chain interruption.”

CHCPs and community leaders emphasized developing or improving infrastructure, such as elevating the CC platform so the CC would not be inundated during floods, repairing the embankment well ahead of time before flooding season starts, and repairing the roads soon after the flood water goes down. One community leader suggested:

“If we had a two or three story building for this clinic, then during the flood we could have uninterrupted health service in the first and the second floor of the clinic, even if the ground floor went under water.”

Community leaders also suggested improved communication between the community, health workers, local administration and the health system. Likewise, an UHFPO suggested implementing a mobile phone text message system to inform the community about service provision capacity of the community clinics during the period of this natural disaster.

Program implementers recommended pre-identification of flood-prone areas and emphasized ensuring that effective advance action plans are in place so that health services can be continued uninterrupted in those areas during flood periods. Since MOH follows routine supply chain procedures for commodity distribution even during the monsoon and flooding months, emphasis was given on offering buffer stock for field level workers before the monsoon starts. Providing additional supplies for the flooding months and post-flood period was also suggested by several categories of respondents.

Another recommendation was to establish an effective referral system by involving community support groups to organize locally available resources like rickshaws or boats to support patients’ travel to referral facilities during floods.

Communities and health care providers highlighted the need for early warning systems, such as risk communication systems to send out messages regarding impending floods and having trained teams equipped with necessary resources to respond and maintain services during and after a natural disaster, like flooding. The CHCPs considered that early warning would help them to communicate with the local stakeholders in advance, to decide on actions in a cohesive manner and to mobilize resources locally to ensure appropriate responses following this type of natural disaster. One UHFPO suggested establishing a special disaster response team to identify immediate needs and to coordinate all the government and non-government responders to work in a coordinated manner.

CHCPs also showed interest in receiving disaster preparedness training. They would then be able to carry out disaster preparedness planning with community members through their community health education sessions.

## DISCUSSION

Our results provide evidence of disrupted but continued iCCM program activities during flooding months in rural communities of Bhola. Even with several constraints and challenges, community level health workers continued offering iCCM services for under-five children within the flood-affected communities. The results demonstrate the potential of continuing community health programming during a flooding disaster. These results align with results from studies conducted in conflict in South Sudan [10] and during Ebola outbreak in Guinea, Liberia, and Sierra Leone [11]. These studies showed that CHWs continued providing iCCM services, although with substantial disruptions in the early emergency periods. All three studies also provide examples of CHWs making ad hoc adaptations to their service delivery in order to adapt to the local context to continue service provision.

These results also echo findings from the studies in South Sudan [10] and Guinea, Liberia, and Sierra Leone [11], as well as findings from the response to Cyclone Nargis in Myanmar [22], showing that supply chain disruptions are a key barrier to continuation of CHW services during a crisis.

Our quantitative data showed increased referrals made by CHCPs and VDs during the flooding months, which was possibly because of medicine shortages. The qualitative data highlighted challenges for the community to comply with referrals, mainly due to lack of availability of transportation and lack of financial resources. CHCPs mentioned their efforts to motivate caregivers/parents to comply with referrals; they also discussed their collaborative efforts with community leaders to arrange local resources and transportation support for families that needed to take their children to referral centers. However, in cases where CHCPs/VDs were out of drugs and referral was not possible, it is likely that ill patients did not receive treatment.

The recommendations of participants at all levels of the health system and in communities highlight the need for improved emergency preparedness. Particularly in this case, where flooding emergencies occur with regularity, instituting a preparedness plan and training key actors on emergency procedures could limit the disruptions in service delivery that were documented here [23]. CHCPs and VDs requested additional training and logistical support to improve services during floods. The key lessons report after Cyclone Nargis in Myanmar also highlighted on importance of training CHWs on disaster response management [22].

There were several limitations of this study. It only included Bhola District of Barisal Division at one point in time. This limits the generalizability of the study, as the duration and intensity of flooding vary by region and from year to year. Infrastructure, workforce, and facilities among health centers may also vary widely throughout the country. The quality of retrieved routine quantitative data from DHIS is unknown. Moreover, we were not able to obtain data from the previous year to be able to assess seasonal trends in the number of treatments provided. Another notable limitation of our study was that we might not have reached the point of saturation with our sample size for qualitative data.

## CONCLUSION

Our study results demonstrate that iCCM services can continue during a natural disaster such as flooding, albeit with significant disruptions. In future emergencies, service delivery could be significantly strengthened by taking key preparedness activities prior to a natural disaster. The following approaches may help to keep iCCM implementation effectively functional during flooding months in similar rural and flood prone areas: 1) identify flood-prone communities; 2) work with health leaders and communities to develop a preparedness plan and procedures for service delivery during flooding emergencies; 3) train community health workers (specifically CHCPs) on the preparedness plan and emergency procedures; 4) establish early warning and response network systems; 5) develop contingency measures to ensure continued supply of essential medicines during emergencies, such as identification of safe storage and delivery of buffer stocks before the flood season; and 6) identify means for facilitation of referrals when road transportation is disrupted.
